# The Role of Family Relationships in Eating Disorders in Adolescents: A Narrative Review

**DOI:** 10.3390/bs10040071

**Published:** 2020-04-02

**Authors:** Michela Erriu, Silvia Cimino, Luca Cerniglia

**Affiliations:** 1 Psychology and Medicine Faculty, Department of Dynamic and Clinical Psychology, Sapienza University of Rome, 00185 Rome, Italy; michela.erriu@uniroma1.it (M.E.); silvia.cimino@uniroma1.it (S.C.); 2 Psychology Faculty, Department of Psychology, International Telematic University Uninettuno, 00186 Rome, Italy

**Keywords:** eating disorders, adolescence, family relationships, family functioning, parental risk

## Abstract

**Background:** Adolescents’ eating disorders have been explored through various conceptual and empirical models. Only recently, scientific literature in this area has more specifically investigated the role of relationships, with particular attention to family functioning. **Objective:** This paper reviews family relationships aspects of eating disorders in adolescence. **Methods:** A narrative literature review of relational issues in adolescents’ eating disorders was performed. **Results:** Empirical evidence of family relationships in adolescents’ eating disorders confirms the relevance of relational aspects in the development and maintenance of the pathology. In particular, the contribution of the relational-systemic approach is wide, suggesting the need to refer to the family context for a better understanding of adolescents’ sufferance. Additionally, the empirical contributions from the conceptual model of Developmental Psychopathology, highlighting the importance of risk and protection factors in family relationships, provides knowledge about the phenomenon of adolescents’ eating disorders in terms of complexity. **Conclusions:** An integrated relational model aimed to explore adolescents’ eating disorders is worthy of investigation to accomplish specific program of intervention.

## 1. Introduction: Eating Disorders in Adolescence

Eating disorders in adolescence are among the most important public health problems in the world [1,2,3,4,5], and they affect a predominantly female population of adolescent girls and young women, from 13 to 25/30 years of age, with a male/female ratio of about 1 out of 10 [6,7,8]. Regarding the adolescent population, epidemiological studies showed a high prevalence of disturbed eating behavior (14–22%), while Anorexia Nervosa, Bulimia Nervosa, and Binge Eating Disorder (BED) were found respectively in 0.3%, 0.9%, and 1.6% of the population [9]. Moreover, 5.7% of females and 1.2% of adolescent males in community samples showed eating disorders, confirming the higher prevalence of the disorder among girls [10].

Defined as body weight control disorders involving significant impairment of physical and psychological functioning, eating disorders represent a category of extremely complex clinical conditions, characterized by abnormal eating patterns, excessive concern for body shape, and abnormal body image perception [6,9]. Due to their complexity, eating disorders can be considered a psychiatric pathology with complex pathogenesis, associated with several individual and relational psychological factors, but also socio-cultural ones. The recent introduction of three specific diagnostic categories, anorexia nervosa, bulimia nervosa, binge eating disorder, in DSM-5 [6] further increases the possibility of describing and understanding in depth the phenomenon of eating disorders. According to DSM-5 criteria, anorexia nervosa is characterized by the restriction in food intake and significantly low body weight, with the intense fear of gaining weight and alteration of the body image. Bulimia nervosa is instead characterized by high food intake in a short time and inappropriate compensatory conduct, such as vomiting, use of purges, and intense physical exercise. Binge Eating Disorder (BED) was included in the DSM-5 as a new nosographic entity, therefore gaining more relevance in the recent extensive revision of the classification of eating disorders. It is characterized by a high amount of food intake without inappropriate compensation behavior and lower interest in weight and body shape compared to Bulimia nervosa.

According to Treasure and colleagues [11], there is a great deal of uncertainty about etiopathogenesis, treatment, and management of eating disorders. It may therefore be useful to report the state of the art on studies on eating disorders in adolescence, mainly focusing on some aspects seemingly going through this type of pathology, although with specificity in different clinical categories.

It is widely posited that adolescence represents a relevant developmental stage during which important changes in social, behavioral, and emotional-motivational functioning occur [12]. For these characteristics, researchers indicate adolescence as a developmental phase at risk for the onset of eating disorders [13,14]. From a relationship point of view, since adolescence represents a period of the life cycle characterized by progressive independence from parents and family members, it may be particularly useful to investigate how family factors provide or do not provide a real basis for the behavioral and emotional wellbeing of the adolescent also with regard to risk conducts. In addition, as most adolescents still live with their parents, the quality of family functioning and the parent–child relationship deserve to be taken into account in relation to offspring’s adaptive development.

In general, the issue of eating disorders in adolescence has been investigated by several theoretical and empirical approaches, among which are psychodynamic models [15], models inspired by Infant Research [16], cognitive models [17,18], and neurobiological models [19]. The majority of these studies have paid much attention to the role of individual variables, whereas little attention has been given to relational aspects linked to the adolescents’ symptoms. In this regard, whether it is clear that eating disorders among adolescents seem to be linked to individual vulnerability, on the other hand, family influence should be recognized as an important element associated with the onset and maintenance of the pathology [20,21,22]. In particular, some relational aspects, such as family functioning and the characteristics and quality of relationships between family members, have gained increasing interest among clinicians and researchers [23,24,25,26,27].

Thus, the heightened scientific attention towards relational and family elements urges summarizing the state of the art of literature in this field.

Based on the above consideration, we intend to discuss the main theoretical frameworks centered upon eating disorders in adolescence from a relational perspective. Moreover, starting from the revision of the primary theoretical contributions on the subject, we also aim to review the empirical international literature on relationships in the family with adolescents with eating disorders. To achieve the purpose of this work, a traditional narrative review was conceived as the most appropriate survey. The narrative review consists of a type of interpretive-qualitative research that can permit descriptively summarizing the results from broad studies on a specific theme. In our opinion, this method is very useful because it can offer the opportunity to deal with different points of view on a relevant topic, increasing the scientific knowledge in a field of study.

## 2. Methods

To carry out the narrative review, we proceeded as follows. The document search was conducted in the electronic databases of Pubmed and Psych INFO. Papers were selected based on the following criteria: Language in English; published in peer-reviewed journals; available abstract. The literature search was undertaken from January 2000 to December 2019. We searched for studies presenting physical, social, and psychological subjects in female adolescents with eating disorders (including anorexia nervosa (AN), bulimia nervosa (BN), and binge eating disorders (BED). The following combined search terms were applied: “eating disorders”, “adolescence”, “family relationships”, and “family functioning”. The title and the abstract of each identified document were examined based on the search strategies. Studies where authors included a sample of adolescents aged between 10 and 24 years of age have only been reported. Moreover, papers in which adolescents were diagnosed with AN, BN, or BED, according to DSM IV ore DSM-5 criteria, were considered. Finally, the thesis dissertations were excluded. 

**Figure 1:**
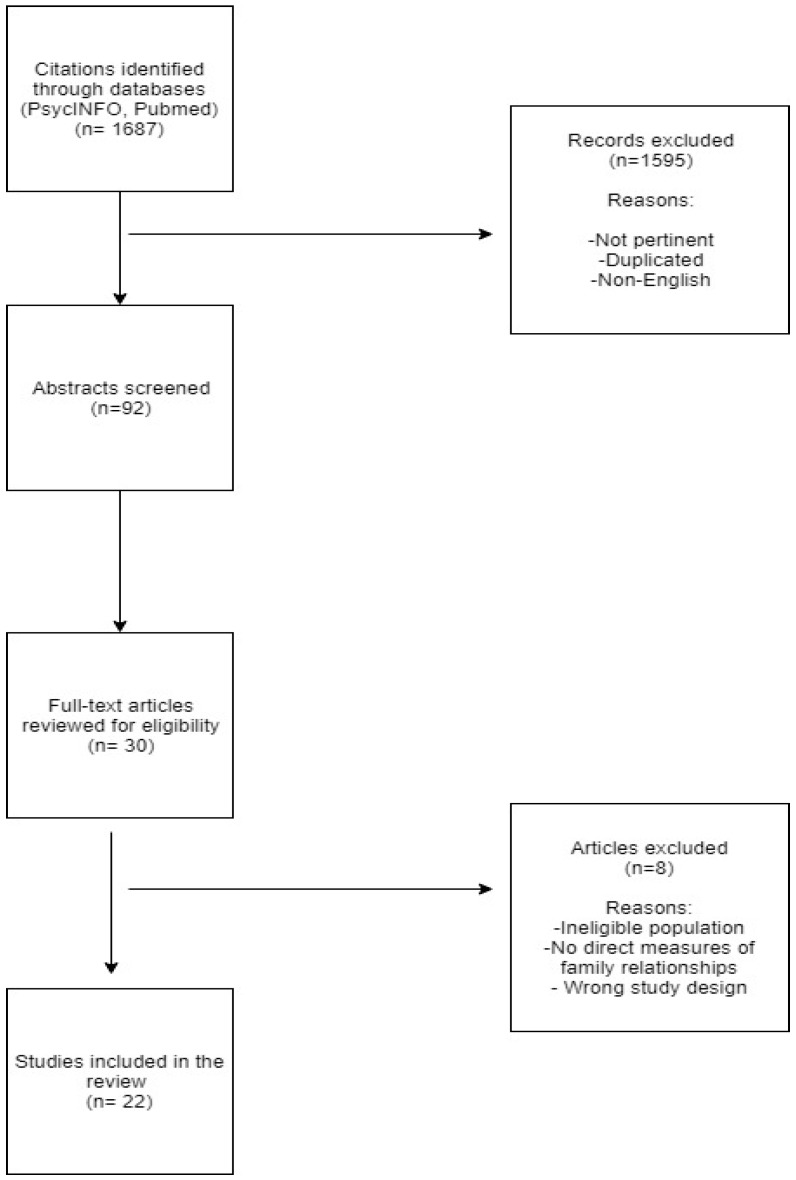
Flow chart.

Originally, we identified a total of 1687 papers on the subject. With respect to preliminary selection of 92 abstracts concerning the above criteria, 22 full texts articles, published in the last 10 years (2009–2019) were selected based on their impact on scientific community (number of citations, impact factor of the publishing journal). The decision to include studies from this decade was based on the fact that our attempt was to collect information on family variables in eating disorders in adolescence through the most recent studies in the international scientific literature that had a focus as consistent as possible with the topic of this review. The flow chart of this narrative review is shown in Figure 1.

## 3. Theoretical Frameworks on Eating Disorders in Adolescence: A Reflection on Relational Aspects

### 3.1. First Theoretical and Clinical Contributions

Originally, the relevance of relational aspects to adolescents’ eating disorders has been acknowledged by a large set of theories and conceptualizations referred to the theory of family systems [28,29,30] and the relational systemic approach [31,32,33,34,35].

#### 3.1.1. The Theory of Family Systems: The Possible Influence of Family Environment on Offspring

The theory of family systems [29,30], which started from the more general theory of systems or systemic theory [36] applied to the study of human problems, conceptualizes the family as a system; that is, as a group of interconnected subjects, whose interactions constitute a "third" reality that does not correspond to the simple sum of the parts (the principle of non-summativity). This model of understanding the family system indicates that the behavior of an individual can be examined only by referring to the family context and therefore to the relationship that the subject establishes with the other members of the family, in particular with parents. According to theorists of family systems, within the family, each individual plays a precise role concerning the other members and for the family as a whole [29]. The central assumption of the theory is that interdependencies between family relationships are influenced by the implicit links and rules for access to resources, materials, and support in the family [29,33]. As a result, family structure can influence individual behavior over time. According to the systemic perspective, the object of study moves from the individual, or from the dyadic mother-child relationship, to the entire system of interactions in which the members of the family nucleus live [29]. Family studies thus oriented have shown that, rather than the individual characteristics or behavioral variables, the dynamic interaction between all the members of the family and the role that each plays in relation to the others can explain the adaptive or problematic development of the members [37]. More in particular, whereas the first theories and clinical research focused on emotional aspects and on the parents expressed affection towards their children, the most recent studies have been interested in the forces among family members that support or affect the offspring’s psychological development [25,37,38,39,40]. Through the notion of the boundary, which indicates the invisible set of expectations that determines the behavior of each member within the family system [41], the theory of family systems identifies the three known profiles related to family interactions (cohesion, disengagement, and entanglement).

In light of this conceptualization of the family system, relationships between family members play a crucial role in the context of the interactions in which the adolescent lives [40,41,42,43]. In fact, in a typical period of the family’s life cycle such as adolescence, family relationships between parents and children can have a significant influence on various aspects of everyday life, including the relationship with the weight and eating routines of offspring. In particular, regarding eating disorders, the theory of family systems identifies relationships between family members as the element that can play a crucial role in the genesis and maintenance of eating disorders [44,45]. As a result, over time, different models of family interactions have been developed, including the systemic-relational approach.

#### 3.1.2. The Relational-Systemic Paradigm: Family Relationships in Eating Disorders

The long tradition of research on eating disorders in the relational systemic field, which begun more than 50 years ago with the pioneering clinical observations of the exponents of the Milan school [34,44,45,46], has highlighted the relationship between the psychopathological aspects of eating and the patient’s family dynamics. In the framework of the theoretical model of family therapy, the two fields represented by systemic relational theories [35,46] and psychosomatic or structural theories [33] have emphasized the transactional modalities of families with problems mainly related to the relationships between the subsystem of children and parents.

Historically, negative family dynamics have been indicated as a key element in the development and maintenance of eating disorders, with initial attention directed toward anorexia nervosa [33,34]. More precisely, the systemic approach postulates that certain types of family organizations are closely related to the development and maintenance of problems in children. In particular, in exploring the theme of dysfunctions in family functioning that can contribute to the onset of an eating disorder, the first systemic conceptualizations of anorexia nervosa in adolescence proposed by the models of psychosomatic families [33] or anorexia nervosa in adolescence [34] have suggested that at the origin of the disorder, there are specific patterns of family interaction and alterations in relationships that could also be determinants in the maintenance and development of the disease. These models essentially originate from clinical observations and lead to a unique description of anorexic families as typically rigid, united, and dedicated to self-sacrifice and loyalty to its members [46]. Mara Selvini Palazzoli, as early as 1963 and 1988 [34,45], focused on the styles and characteristics of the parental couple. The author describes a family organization characterized by an intrusive, intolerant, and hypercritical mother and by a father who is often brilliant but absent from the family. In this type of environment, where there is no real emotional support, the patient expresses her discomfort through an abnormal and provocative eating behavior, which on the one hand allows her to obtain a form of control and self-affirmation in her relationship with her family, but on the other hand leads her to a further confirmation of her position of dependence and loneliness [47,48].

Selvini Palazzoli [35] also takes into consideration the communicative styles and interactive models of families with anorexic daughters, through a constant work of redefining emotions from the outside, which are not only denied, but disconfirmed. According to these relational patterns, each member of the family acts in relation to the needs of others and for the good of someone else. The author speaks of a “three-way marriage” where it is as if each member is married to two people: The father with the mother and daughter, and the daughter with her father and mother. All this will not allow the daughter to acquire real independence and to live an autonomous life. In the same years as the studies of the exponents of the school of Milan, Minuchin and colleagues [33] introduced the role of the family in eating disorders by conceptualizing a new model of anorexia nervosa, called the “psychosomatic family”. The author speaks of anorexia as a psychosomatic syndrome, characterized by symptoms of both a physical and psychological nature, which develops in certain family organizational contexts. The daughter who suffers from anorexia nervosa is the bearer of a malaise that is not only personal, but also of the family and, in a broader sense, social. Symptoms play an important role in the maintenance of family homeostasis, since on the one hand, they represent the protest of the daughter against the family system, configuring itself as an attempt to break up the status-quo and pseudo-individuation, but on the other hand they result in keeping the daughter in a condition of dependence on her parents and therefore of the impossibility of change. In this context, eating disorders are seen as part of and the result of a pathological pattern of interaction between the subject and family [41]: According to this perspective, we talk about “family psychosomatic”.

## 4. Empirical Evidence of the Family Relationship in Eating Disorders: Synthesizing the Contributions

The narrative review we propose will analyze the documents published in the most recent databases that have been taken into account family relational issues in adolescents’ eating disorders. As suggested by previous literature [48,49], in Table 1, we report the main characteristics of included studies.

### 4.1. Results 

#### 4.1.1. Contemporary Approaches

The descriptions of eating disorders in the family with adolescents given by clinicians and pioneers of family therapy have contributed to providing a general model of this pathology [68]. Yet, as Dare and colleagues point out in their contribution [69], the first systemic theories were primarily based on subjective clinical observations and not empirically founded. The analysis of the articles shows that only in the last decades the researchers have been investigating the relationships in families with eating disorders more systematically. Consistently, some authors have focused on family interactions in families with children with eating disorders, compared to families with children without a diagnosis of eating disorders [50,69]. On the other hand, some authors have tried to identify specific family interactional patterns related to the diagnosis of eating disorders [50,51]. More specifically, some studies have examined the differences in family functioning between families with eating disorders and non-clinical families. These studies have reported that by considering only family functioning, in general, the family functioning in the clinical groups is lower than in the control groups [70,71]. In contrast, when the different components of family functioning, such as emotional involvement, communication, and organization are considered, the results are variable and in some samples contradictory [71]. Additionally, with regard to conflict, while some studies have reported high levels of conflict in clinical samples [72], other studies have indicated no significant differences between clinical groups and control groups, and other papers have shown lower levels of conflict in clinical samples [73]. This early empirical evidence is very interesting because they provide a complex picture of family relationships in eating disorders that are difficult to delineate univocally. Indeed, although the reported worse family functioning is a crucial element in families with eating disorders, compared to control families, there is no empirical evidence of a specific dysfunctional pattern for families with eating disorders, even more so when referring to the different diagnostic categories of eating disorders [25]. Even in the most recent studies, results concerning the evaluation of family functioning among the different diagnostic categories of eating disorders are divergent. Some studies have shown no significant differences between the diagnostic categories of eating disorders [74,75]. Other studies have found significant differences between the types of eating disorders in relation to different elements of family functioning: For example, cohesion and orientation to achievement were found to be worse in families with anorexia, compared to bulimic families [73]. Still, other empirical evidence from studies based on the hypotheses of the pioneers of family therapy has highlighted that families with eating disorders may show excessive cohesion and lack of flexibility, with a profound lack of emotional expression [76]. Furthermore, recent studies have partly confirmed the clinical observations of family therapists, highlighting that parents and adolescent daughters with eating disorders show conflicting interactions, characterized by little reciprocity and little emotional harmony [77]. These patterns of interaction are consistent with recent empirical evidence on the poor quality of child interactions during childhood, as reported by some authors [39,52]. In this regard, Hayaki [78] highlights that parental intrusiveness and/or withdrawal in exchanges with children in the first five years of life can facilitate the onset of eating disorders of daughters during adolescence, especially when parents have experienced traumatic events and physical and psychological abuse in their own lives [39]. Since many studies have shown an association between the family context in general and eating disorders, conclusions about the causal role of family environment in eating pathology cannot be drawn.

It may be noted that nowadays, current international scientific literature rejects the “causal family notion”, that is to say, the idea that family is the only cause (in etiological terms or the main risk factor) of eating disorders [40]. In support of this, the Academy for Eating Disorders [79], states that any generalized model of an eating disorder that includes the family as the primary cause must be rejected since it inevitably implies blaming the parents for the disease of their children. According to this position, the directions from complexity theories [36] and from Developmental Psychopathology [80] suggest the etiopathogenesis of eating disorders should be considered a multifactorial cause, linked to genetic, psychological, neuro-endocrine, sociocultural, and family factors [81]. Yet, the effort of empirical research in the field of family processes and adolescents’ psychopathology continues. In particular, international literature has focused on the role of family factors in the development and maintenance of eating disorders in offspring. In this respect, there is a general agreement about the important role of family environment on children’s eating, particularly in terms of organizing physical activities and managing behaviors related to wellbeing or weight control, such as diets and restrictive diets [82]. Research has shown that, together with parental behavior, family functioning is linked to the wellbeing of adolescent children [53]. At the same time, however, only a few studies have investigated the influence of family factors such as family functioning or the quality of the adolescent parental relationship on problematic behavior and eating disorders in offspring [83].

#### 4.1.2. An Empirical Relational-Systemic Perspective of Family Functioning

If, as we have seen, systemic studies have only highlighted the role played by family in eating disorders in adolescence from a clinical point of view, recently, thanks to the studies of Olson and colleagues [54,84,85,86,87], an empirical approach to the theme of family functioning takes form, with the aim to operationalize the notions already known in clinical practice. In recent years, in particular, empirical research in the systemic-relational field has sought to further explore family functioning as a possible variable in the onset and maintenance of the disorder [55,88]. It is necessary to remark that the Olson Circumflex Model of Conjugal and Family Systems [84] represents one of the most widely used international models for assessing family functioning (as it is perceived by the members of the system) and it is based on the three dimensions of cohesion, adaptability, and communication. Recent studies focused on risk factors in the development of eating disorders have confirmed that family functioning plays an important role in the assumption and maintenance of dysfunctional eating behaviors in adolescence [56,74,89]. An important point in this regard is that the development of eating disorders in adolescence seems to be influenced by the perceptions of family members regarding family functioning, particularly concerning family cohesion, adaptability, and communication [56,90]. Some empirical piece of evidence has indicated, in particular, that families with poor cohesion, low affective expression, and excessive interpersonal dependence among members were found to have a higher risk of developing pathological eating behavior [56,57,90,91]. In addition, research has shown that adolescents with an eating disorder report high levels of family dissatisfaction [92,93]. In particular, adolescents with eating disorders were found to experience unsatisfactory family relationships, characterized by poor parental acceptance (family warmth, empathy, emotional support) and limited independence among members [58]. On the contrary, adolescents who report emotional support by their parents seem less likely to develop an excessive concern about weight, body dissatisfaction and high ideals of thinness, and to assume bulimic behavior [59]. Thus, in general, high family unity protects adolescents from emotional stress and is associated with adaptive behavior [94]. The research, in this sense, well documents the relationship between positive family functioning and healthy eating behavior, identifying the positive perception of family relations as an important protective factor for the risk of developing disturbed eating behavior [60,95].

The development of eating disorders seems to also be influenced by the assumption of binding and unfavorable family rules [73]. Families with eating disorders were found to have special rules regarding the restriction of thoughts, feelings, and self: Family rules related to the prohibition to discuss, solve problems, and talk about circumstances that could cause discomfort in the family, represent an index of family rigidity; rules related to mutual support, sharing of decisions to be taken, and emotional ties, however, would facilitate family cohesion [96]. In particular, families with strict family rules that hinder or restrict the expression of thoughts, feelings, and self (prohibition to configure and talk about situations that can cause discomfort) are exposed to a risk for the development of eating disorders, especially in cases where restrictive rules concern food [73]. Also, a critical attitude and family pressures regarding the body and physical form constitute indices of involvement and family rigidity, which strongly affect the development of the disease, facilitating the assumption of inadequate eating habits [97,98,99]. Also, weight concerns and stereotypes of female beauty portrayed in the media and the uptake of diets predict an increased risk of developing a binge eating disorder in the adolescent and pre-adolescent girls [100]. 

In Italy, several studies using the recent version IV of FACES questionnaire [87] for the evaluation of family functioning and communication in families with eating disorders have confirmed the main structural and functional characteristics of the anorexic family indicated by Minuchin [61,101]. Visani and colleagues [88] have investigated the differences in family styles among families with anorexic and bulimic eating disorders, highlighting the heterogeneity in development pathways; the authors have reported that families with anorexia nervosa show problematic scales of FACES IV but also discrete protective factors, unlike families with bulimia nervosa, characterized by low levels of protective factors of cohesion and flexibility and a high level in disorganization and disengagement. In general, compared to families with children without eating disorders, families with patients with eating disorders report worse family functioning, although it is still unclear whether there are specific differences in relation to the different diagnostic typologies [25]. For example, studies on the evaluation of the representations of family functioning by adolescent females with eating disorders, using semi-clinical tools (such as interviews), have confirmed that bulimic subjects tend to describe their families as highly conflictual, while anorexic patients report their families as cohesive and well organized, but with little tolerance to conflict [24]. As for the perception of family functioning in binge eating disorder, adolescents with this diagnosis report a negative perception of family functioning in terms of dysfunctional family interactions, characterized by a poor expression, problematic communication, poor cohesion and affectivity, and greater expression of conflict [102].

With regard to perceived family functioning between different family members, many studies have shown disagreement among family members. In particular, some studies have found significant differences between the views of patients with eating disorders and those of one or both parents [62]. Parents of patients with eating disorders, moreover, particularly those with anorexia nervosa, have reported increased family conflicts and feelings of stress and depression [51]. It remains unclear whether and how the perceptions of mothers differ specifically from those of fathers [24]. However, patients were found to perceive worse family functioning than their parents. In addition, when family functioning perceived by both parents was considered, parents reported their family as significantly more cohesive and with high emotional expression, while patients reported their family as highly conflictual [103,104]. Still, considering the assessments of mothers and fathers separately, some studies have shown that children describe their family as problematic in terms of general functioning, communication, and problem-solving, compared to both parents [105]. Moreover, some studies have shown that anorexic patients describe their family as more dysfunctional than mothers do [7], and other studies have highlighted the difference in family perception among all members concerning adaptability [106]. 

Finally, comparing the perspective of mothers with that of fathers, in anorexic families, mothers and children are more dissatisfied with their family than fathers [106] while other studies have shown a significant difference between the point of view of mothers and fathers in relation to emotional responses and problem-solving [72,73]. In an interesting recent study, in which children diagnosed with eating disorders and their parents completed the version IV of the FACES tool, Fisher and Bushlow [103] have found that most patients and parents represent their families as connected/very connected and flexible/very flexible, with moderately low levels of entanglement, rigidity, chaos, moderate levels of communication, and low levels of satisfaction.

#### 4.1.3. A Possible Integrate Perspective for Understanding Eating Disorders: Developmental Psychopathology and Relational Systemic Approach

As seen until now thanks to the contribution of empirical studies on family factors in eating disorders, in particular, those that have used the FACES-IV instrument [85,86,87] family models multi-determined and contextual psychopathological processes in developmental age is gaining ground. All the results of the studies indicate a shift from a focus on family deficits towards family resources, which is a gradual encouragement of family skills and positive connections between members [74].

According to Cook-Darzens and colleagues [72], rather than talking in terms of dysfunctional families, it is more appropriate to consider the families with eating disorders as a system trying to function with different styles and levels of adaptation (fit) in relation to specific modes of internal organization and coping strategies. Thus, the already innovative suggestions in the study of dysfunctional family functioning proposed by the systemic-relational approach can be further enriched by referring to the Developmental Psychopathology [104], a new discipline that places relationship among its crucial principles, overcoming the idea of mental disorder as an individual problem and emphasizing the importance of risk factors within family relationships. With regard to family relationships, studies in the area of Developmental Psychopathology have shown that a lack of parental sensitivity can interfere with the ability of adolescents to regulate their behavior and emotions [63,107,108,109]. In addition, other studies have indicated that experimenting problematic family relationships and inadequate parenting behavior (such as undemocratic parenting, lack of interest in children’s lives, poor promotion of autonomy, poor emotional involvement) can influence the appearance of body dissatisfaction in adolescents [64] and the assumption of problematic eating behavior [110]. Several recent studies have also shown an association between eating disorders in female adolescents, maternal psychopathology and/or psychopathological risk (anxious and depressive symptoms, eating disorders) [111], and problematic family functioning (poor quality of relationships between family members). An important aspect to underline is that, generally, the international scientific literature referred to Developmental Psychopathology has indicated maternal psychopathology as the main risk factor for the development of emotional–behavioral problems in children [112,113,114,115]. For instance, recent studies have found that poor maternal emotional regulation can lead to problems in family functioning due to distancing or excessive responsiveness. In particular, difficulties in maternal emotional regulation in families with adolescents could lead to increased risk for children in this particular stage of development [65,66]. Moreover, a recent study has pointed out that difficulties between parents and children involve negative psychological consequences for adolescents [43].

Little scientific attention to paternal psychopathological profiles has been given. This gap in the international literature on the family dynamic in eating disorders seems to be very relevant since fathers can represent an important risk factor or protection/mitigation for offspring’s wellbeing. Only recently have some authors have paid attention to the father figure in offspring’s eating disorder, offering important elements for a systemic interpretation of adolescents’ psychopathological functioning [67,116,117,118].

## 5. Conclusions

The present review aimed to provide a summary of the scientific research findings on relationships in adolescents’ eating disorders. The analysis of the literature we performed found that, originally, the relevance of relational aspects to adolescents’ eating disorders has been acknowledged from a clinical perspective and different theoretical framework was adopted to explain the family dynamics in adolescents’ disease [29,32,33,34,35]. Afterward, the role of family functioning in eating disorders has been empirically supported in greater depth [69,71,73,106]. Finally, more recently, theoretical and empirical research has begun to converge towards integrated multifactorial models of the family with eating disorders, in which the relationship between parents and adolescents results as crucial [118]. Moreover, even though the interest of clinical and researchers in the area of adolescents’ psychopathology has turned to the role of parental profiles, only a few studies, to our knowledge, have investigated the father figure [27,67,116].

In general, it is important to note that, although a great deal of studies has generally highlighted the relevance of family relationships in the offspring disease, results concerning the role of family factors are so divergent that, nowadays, definitive statements about direct causation cannot be drawn [40,119]. Our review has found that some but not all results of the studies on families with eating disorders members support the notion of the psychosomatic family described by Minuchin (entangled hyper-protective, rigid, conflict-avoiding, and with poor problem-solving capacity). Thus, nowadays, no substantial data confirm an empirical model of psychosomatic families, as conceptualized by the pioneers of family therapy [33,34,35].

In support of the difficulty to empirically confirm the theories of the pioneers of family therapy, some studies show that even patients with psychiatric diagnoses different from eating disorders, such as obsessive-compulsive disorder and anxiety disorder [117], and their families report a problematic family functioning, which can therefore not be indicated as a unique and peculiar feature of eating disorders [116]. Furthermore, the empirical evidence reported shows that families with eating disorders vary in their family functioning, presenting strong discrepancies in the representation of relationships by its members. In brief, the areas of family malfunctioning identified are so varied that it is not possible to identify, with certainty, either the family structure or, above all, the dysfunctional patterns typical for the populations of the different eating disorders. In our opinion, the integration of the relational systemic approach and Developmental Psychopathology sheds light on a promising research scenario. We strongly believe that an integrated approach may be the appropriate survey to conceptualize human functioning in systemic terms, as promoted by the pioneers of family therapy. Indeed, the results of this review, together with other studies in the literature, point out the need to refer to human functioning through a key lecture of complexity and multi-determination. Considering the contribution of different factors of vulnerability, both individual and environmental, in the investigation of the development of pathological trajectories, is crucial [80].

As a whole, this review highlights the relevance of family factors in the onset and maintenance of eating disorders. In particular, the presence among family member of general positive affective relationships represents a central aspect for the emotional wellbeing of the subjects, especially in adolescence [82]. Understanding the role of relational elements, particularly communicative ones, between parents and adolescents can greatly help to investigate such an important period of life as the adolescence. In this sense, this study provided information about the factors of resilience and vulnerability of adolescents, with respect to eating behavior but also to the general adolescents’ emotional adaptive psychological functioning. Thus, our findings can be used not only to understand the adolescents’ disease, but also to support all families that deal with difficulties with their offspring.

Some limitations of this review warrant further attention in future studies. In particular, our results should be viewed in the light of several methodological limitations. For example, possible differences in the relation between family functioning and eating disorders in function of the age of the offspring have been not deepened. Moreover, this study did not consider possible differences according to the family structure (e.g., single parents) or the relevance that relationships with peers and romantic partners may have during adolescence. Hence, in order to have a more complete understanding of eating disorders, future studies should continue investigation of these aspects.

With this work, we hope to contribute to ponder over the complexity of the pathology in developmental age and to improve the knowledge of all the aspects that must be taken into consideration when dealing with adolescents’ sufferance. Multiple parts in adolescents’ problems are involved, such as clinical, researchers, and overall familiars, so that an integrated relational model for exploring adolescents’ eating disorders is worthy of attention, not only to understand, but also to carry out specific programs of intervention.

## Figures and Tables

**Table 1:** Characteristics of included studies.

Study	Year	Design	Country	Sample Size	Type of Sample	Age Range (Years)	Investigated Family Issues	Main Results
Berge et al. [22]	2014	C	USA	N = 2793	R	11–19	Family functioning and parenting practices	Parental psychological control moderated the protective relationship between family functioning and disordered eating behaviors in adolescent girls
Berge et al. [23]	2010	C	USA	N = 2516	R	13–18	Family relationships and parental style	Authoritative parenting style may play a protective role related to adolescent overweight
Aragona et al. [36]	2011	C	Italy	N = 60	CL	13–18	Family functioning (adaptability and cohesion) and psychopathological symptoms	High levels of cohesion were found in families with adolescents with eating disorders (hyper-involvement of family members)
Leung and Shek [42]	2014	C	China	N = 275	HR	11–16	Family relationships (parent–adolescent; parental responsiveness and control)	Adolescents generally perceived lower levels of parenting behaviors than did their parents
Gillett et al. [50]	2009	C	USA	N = 102	CL	14–20	Family process rules (kindness; expressiveness and connection; constraining thoughts, feelings, and self; inappropriate caretaking; and monitoring	Eating-disordered youth reported a lower proportion of facilitative family rules and a higher proportion of constraining family rules than did parents and siblings
Sim et al. [51]	2009	C	USA	N = 55	CL	14–18	Family functioning and psychological symptoms	Families of girls with AN experienced greater family conflict, reduced parental alliance, and increased feelings of depression
Hayaki [52]	2009	C	USA	N = 115	HR	16–20	Family situation and emotion dysregulation (alexithymia and experiential avoidance)	Individuals who expect eating to provide emotional relief may be especially susceptible to disordered eating (bulimia nervosa)
Haines et al. [53]	2016	C	Canada	N = 3768	R	14–24	Family functioning and quality of mother- and father-adolescent relationship	High family functioning was associated with lower odds of disordered eating
Tafà et al. [54]	2017	C	Italy	N = 90	CL	13–15	Family functioning (adaptability and cohesion) and psychopathological symptoms	Anorexic families show a maladaptive functioning and anorexic adolescents present intense psychopathological disturbances
Visani et al. [55]	2014	C	Italy	N = 35	CL	14–17	Family functioning (adaptability and cohesion) and psychopathological symptoms	Families with female adolescents with eating disorders report a problematic family functioning, with anorexic daughters showing severe psychopathological symptoms
Lyke and Matsen [56]	2013	C	USA	N = 91	R	14–18	Family functioning (problem-solving, communication, roles, affective involvement, or behavior control)	Unhealthy general functioning predicted adolescent problems
Goossens et al. [57]	2012	L	Belgium	N = 601	R	10–12	Parent-child relationship (parental style and attachment)	Longitudinal association between parent-child relationships and eating pathology and weight gain in preadolescents.
Laghi et al. [58]	2012	P	Italy	N = 438	R	14–18	Family functioning (adaptability and cohesion) and psychopathological symptoms	Family functioning predicts risk factors of eating disorders (binge eating disorder)
Hasenboehler et al. [59]	2009	C	Switzerland	N = 57	R	10–12	Family structures (hierarchy, conflict, restrained eating)	Family structure is associated with overweight and with eating behavior
Neumark-Sztainer et al. [60]	2009	L	USA	N = 412	HR	14–18	Family structures (family connectedness, body satisfaction, regular meals)	Family connectedness represents a protective factor for disordered eating among overweight adolescents
Laghi et al. [61]	2017	C	Italy	N = 72	CL	Mean age 14.86 years	Family functioning (adaptability and cohesion)	Girls with anorexia nervosa poor satisfaction about family environment and rated their families as less communicative, flexible, cohesive, and more disengaged
Fisher and Bushlow [62]	2015	C	USA	N = 44	CL	14–18	Family functioning (adaptability and cohesion)	A great majority of patients and parents reported their families as being connected/very connected
Haycraft et al. [63]	2014	C	UK	N = 528	R	13–15	Family situation (perceptions of parental feeding practices)	An intense perceived pressure from parents to eat food and lower perceived parental responsibility for food are related to more unhealthy eating-related attitudes in female adolescents
Micali et al. [64]	2014	L	UK	N = 7082	R	13–15	Family burden and psychological symptoms	An extreme level of fear of weight gain, avoidance of fattening foods, and distress about weight and shape were common among girls
Horesh et al. [65]	2015	C	Israel	N = 86	R	13–16	Parent-child relationship (father-daughter relationship; parental style: bonds and protection)	A negative perception of the father’s parenting style is associated with eating disorders and depressive symptoms
Pilecki and Józefik [66]	2013	C	Poland	N = 112	CL	13–20	Intergenerational family relationship (autonomy, intimacy)	A relevant association between daughters’ and fathers’ perceptions of autonomy in their families of origin was found (transgenerational transmission of autonomy and intimacy in eating disorders)
Ciao et al. [67]	2015	P	USA	N = 80	CL	15–18	Family-based treatment and supportive psychotherapy	Treatments were found to be efficacious with respect to bulimic symptoms

**Notes:** Design: L = Longitudinal study; C = Cross-sectional study; P = Prospective. Type of sample: R = Representative; CL = Clinical; HR = High Risk.
